# Self-Similarity and Power-Law Spectra of Polymer Melts and Solutions

**DOI:** 10.3390/polym14193924

**Published:** 2022-09-20

**Authors:** Jehyeok Choi, Kwang Soo Cho, Mi Kyung Kwon

**Affiliations:** 1 Department of Polymer Science and Engineering, Kyungpook National University, Daegu 41566, Korea; 2 Division of Biotechnology, DGIST, Daegu 42988, Korea

**Keywords:** self-similarity, relaxation time spectrum, dimension analysis, linear viscoelasticity

## Abstract

Both the Rouse and Doi-Edwards models can be expressed by the relaxation spectra, in the form of power-law functions. The concept of self-similarity has offered a simple solution to many problems in polymer physics. Since the solutions derived from self-similarity are power-law functions, it is essential to check whether the relaxation spectrum of polymeric fluids can be derived by self-similarity. In this study, the power-law spectrum of an unentangled polymer solution is derived by using the self-similarity approach, which does not work for entangled polymeric fluids. Although Baumgaertel et al. (Rheol. Acta 29, 400–408 (1990)) showed that the power-law spectrum can quantitatively describe the linear viscoelasticity of monodisperse polymer melts, regardless of molecular weight, they did not find the universality of the exponent of the spectrum because they found different exponents for different polymers. Under the consideration existing the universality of linear viscoelasticity of polymer melts, this paper deals with the universality of the exponent by employing a new regression algorithm and confirms that the exponent is independent of the type of polymer.

## 1. Introduction

Self-similarity has achieved remarkable achievements in solving many problems in polymer physics. Although the scaling concept has been applied to the linear viscoelasticity of polymer solutions and melts [1], self-similarity has not been used for the relaxation time spectrum of polymeric fluids. 

The mathematical results of the application of self-similarity are usually expressed by power-law functions [2,3]. If polymeric fluids obey self-similarity, it is natural that their relaxation time spectrum should be expressed by a power-law function. Without considering self-similarity, Baumgaertel et al. suggested the power-law spectrum to describe the linear viscoelasticity of nearly monodisperse polymer melts [4]. The power-law spectrum agreed quantitatively with the experimental data. However, the power-law spectrum adopted by the authors had different exponents for different types of polymers. It is, thus, questionable whether the power-law spectrum can be derived from self-similarity analysis. Another question surrounding the success of the power-law spectrum pertains to the universality of the exponent of the spectrum. Cho et al. showed that the linear viscoelasticity of monodisperse polymer melts can be superposed on a single curve by dimensional analysis, which seems to support the universality of the exponent [5].

This study comprises two parts, containing answers to two questions. In Section 2, we will derive the power-law spectra for unentangled polymer solutions through self-similarity analysis and show the failure of self-similarity in entangled polymeric fluids. In Section 3, we will reveal the universality of the exponent for the experimental data of four monodisperse polymers. In order to show universality, we need to introduce a special nonlinear regression algorithm.

## 2. Self-Similarity Analysis

### 2.1. Simple Molecular Theories

The Rouse model was the first molecular theory introduced for unentangled polymeric fluids [6]. The relaxation modulus of the Rouse model is defined as

(1) $$G_{\mathrm{Rouse}}\left(t\right)=G_{R}\sum_{p=0}^{\infty }\exp\left(-\frac{p^{2}t}{\lambda_{R}}\right)$$

where t is the time, $G_{R}$ is the scale factor of the relaxation modulus, and $\lambda_{R}$ is the Rouse relaxation time. The summation can be approximated by the following integral:

(2) $$G_{\mathrm{Rouse}}\left(t\right)\approx \int_{0}^{\infty }G_{R}\exp\left(-\frac{p^{2}t}{\lambda_{R}}\right)dp=\int_{-\infty }^{\infty }\frac{G_{R}}{2}\sqrt{\frac{\lambda_{R}}{\lambda }}\exp\left(-\frac{t}{\lambda }\right)d\log\lambda$$

where $\lambda =\lambda_{R}p^{-2}$. From Equation (2), the relaxation time spectrum of the Rouse model, $H_{\mathrm{Rouse}}\left(\lambda \right)$, can be derived as follows:

(3) $$H_{\mathrm{Rouse}}\left(\lambda \right)=\frac{G_{R}}{2}{\left(\frac{\lambda }{\lambda_{R}}\right)}^{-1/2}.$$

This is the power-law spectrum, with the exponent −1/2. 

For entangled polymer melts, Doi and Edwards [7] provided the relaxation modulus, such that

(4) $$G_{\mathrm{DE}}\left(t\right)=G_{e}\sum_{p=0}^{\infty }\frac{1}{{\left(2p+1\right)}^{2}}\exp\left[-\frac{{\left(2p+1\right)}^{2}t}{\lambda_{D}}\right].$$

A similar method derives the following power-law spectrum:

(5) $$H_{\mathrm{DE}}\left(\lambda \right)=\frac{G_{e}}{4}{\left(\frac{\lambda }{\lambda_{D}}\right)}^{1/2}$$

where $G_{e}$ is the plateau modulus as a scale factor, and $\lambda_{D}$ is the reptation relaxation time of the Doi-Edwards model. It is interesting to note that the relaxation time spectrum of the Doi–Edwards model is an increasing function of λ. Thus, we need to find the upper bound of relaxation time for the power-law spectrum.

With the help of two simple molecular theories, we considered a general form of the power-law spectrum, with μ as an exponent, as follows:

(6) $$H\left(\lambda \right)=H_{c}{\left(\frac{\lambda }{\lambda_{\max}}\right)}^{\mu }$$

where $H_{c}$ is the scale factor of the spectrum with the dimension of modulus, and $\lambda_{\max}$ is the maximum relaxation time. $\lambda_{\max}$ corresponds to $\lambda_{R}$ for the Rouse model, with $\lambda_{D}$ for the Doi-Edwards model. The assumption of the existence of maximum relaxation time leads to the following:

(7) $$\begin{array}{l} G\left(t\right)=\int_{0}^{\lambda_{\max}}H_{c}{\left(\frac{\lambda }{\lambda_{\max}}\right)}^{\mu }e^{-t/\lambda }\frac{d\lambda }{\lambda };\\ G^{\prime }\left(\omega \right)=\int_{0}^{\lambda_{\max}}H_{c}{\left(\frac{\lambda }{\lambda_{\max}}\right)}^{\mu }\frac{\lambda^{2}\omega^{2}}{1+\lambda^{2}\omega^{2}}\frac{d\lambda }{\lambda };\\ G^{\prime \prime }\left(\omega \right)=\int_{0}^{\lambda_{\max}}H_{c}{\left(\frac{\lambda }{\lambda_{\max}}\right)}^{\mu }\frac{\lambda \omega }{1+\lambda^{2}\omega^{2}}\frac{d\lambda }{\lambda } \end{array}$$

where ω, $G\left(t\right)$, $G^{\prime }\left(\omega \right)$, and $G^{\prime \prime }\left(\omega \right)$ are the frequency, relaxation modulus, storage modulus, and loss modulus, respectively. 

It should be noted that, if μ>0, $G^{\prime \prime }\left(\omega \right)\propto \omega^{-\mu }$ for $\lambda_{\max}\omega >1$. Since most monodisperse polymer melts show $G^{\prime \prime }\left(\omega \right)\propto \omega^{-1/4}$, the original Doi-Edwards model had some discrepancy with the experimental data. However, Doi showed that the introduction of contour length fluctuation gives μ=1/4 [8]. Baumgaertel et al. [4] suggested that μ≈0.22 for polystyrene, and μ≈0.42 for polybutadiene. It should also be noted that Baumgaertel et al. [4] determined the exponent from the discrete spectrum, which was calculated by their algorithm, called IRIS [9]. Although IRIS is an excellent algorithm, the discrete spectrum is not unique, and it is an approximation of the continuous spectrum, which is mathematically unique. Therefore, the universality of μ should be checked for various kinds of polymers.

Incidentally, Equation (6) tells us that dimensional analysis and self-similarity analysis can be used to determine the two parameters $H_{c}$ and $\lambda_{\max}$.

### 2.2. Self-Similarity Transform Invariance 

The core of the scaling concept is that measurable quantities of polymer physics do not change, even if the original monomer is replaced by an equivalent monomer, which is a collection of α original monomers. The introduction of an equivalent monomer changes the number of monomers by $N\to \alpha^{-1}N$ and size of the monomer by b→βb, where β is unknown. This microscopic transform should not alter the size of the polymer chain $R=bN^{\nu }$, where ν is 1/2 without the excluded volume effect and about 3/5 with the excluded volume effect. This helps in representing β, in terms of α: $\beta =\alpha^{\nu }$.

Consider a macroscopic quantity A, which is a function of N, b, the volume fraction of the polymer (ϕ), and so on. Dimensional analysis gives rise to

(8) $$A=A_{c}\Pi \left(\tilde{x}\right)$$

where $A_{c}$ is the scale factor of A, and $\tilde{x}$ is a dimensionless variable, which is a function of N, b, and ϕ. $\Pi \left(\tilde{x}\right)$ is a dimensionless function. If the quantity obeys self-similarity, we can consider the following self-similarity transform:

(9) $$A\to \alpha^{0}A;\;A_{c}\to \alpha^{\gamma }A_{c};\;\tilde{x}\to \alpha^{\tau }\tilde{x}$$

where the transform exponents γ and τ can be determined if we know the function structures of $A_{c}$ and $\tilde{x}$, respectively. We can then use

(10) $$N\to \alpha^{-1}N;\;b\to \alpha^{\nu }b;\;\phi \to \alpha^{3\nu -1}\phi .$$

Equation (8) can, thus, be rewritten as

(11) $$A_{c}\Pi \left(\tilde{x}\right)=\alpha^{\gamma }A_{c}\Pi \left(\alpha^{\tau }\tilde{x}\right).$$

Since $A\to \alpha^{0}A$, Equation (11) must be independent of α. Differentiation of Equation (11), with respect to α, gives

(12) $$\gamma \alpha^{\gamma -1}\Pi \left(\alpha^{\tau }\tilde{x}\right)+\tau \alpha^{\gamma +\tau -1}\tilde{x}\frac{d\Pi }{d\left(\alpha^{\tau }\tilde{x}\right)}=0$$

Equation (12) gives

(13) $$\frac{d\log\Pi }{d\log\tilde{x}}=-\frac{\gamma }{\tau }.$$

This means that $\Pi \left(\tilde{x}\right)\propto {\tilde{x}}^{-\gamma /\tau }$, which is a power-law function. We can, thus, express the quantity A analytically.

However, if γ=τ=0, we cannot specify the dimensionless function $\Pi \left(\tilde{x}\right)$ if additional information is unavailable. In the power-law spectrum of Equation (6) mentioned earlier, the scale factor $H_{c}$ can be formulated by an invariant of the self-similarity transform, and so can $\lambda_{\max}$. However, due to the lack of information, we could assume the mathematical form of relaxation time spectrum, Equation (6). We will later reveal the additional information allowing us to determine the exponent μ.

### 2.3. Scale Factors

We need to determine $H_{c}$ and $\lambda_{\max}$ before determining μ. For the polymer solution, one can consider

(14) $$H=H\left(k_{B}T,\;\phi ,\;b,\;N,\;\lambda ,\;\eta_{s}\right)$$

where $k_{B}$ is the Boltzmann constant, T is the absolute temperature, and $\eta_{s}$ is the viscosity of the solvent. Dimensional analysis gives

(15) $$H=H_{c}\Pi \left(\frac{\lambda }{\lambda_{c}},\;N,\;\phi \right)$$

where

(16) $$H_{c}\equiv \frac{k_{B}T}{b^{3}};\;\lambda_{c}\equiv \frac{\eta_{s}b^{3}}{k_{B}T}.$$

According to the Buckingham theorem, we need three dimensionless variables, such as $\lambda /\lambda_{c}$, N, and ϕ. We can then derive the following self-similarity transforms:

(17) $$H_{c}\to \alpha^{-3\nu }H_{c};\;\lambda_{c}\to \alpha^{3\nu }\lambda_{c};\;N\to \alpha^{-1}N;\;\phi \to \alpha^{3\nu -1}\phi .$$

Application of Equation (17) to Equation (15) gives

(18) $$\alpha^{-3\nu }\Pi \left(\alpha^{-3\nu }\frac{\lambda }{\lambda_{c}},\;\alpha^{-1}N,\;\alpha^{3\nu -1}\phi \right)=\Pi \left(\frac{\lambda }{\lambda_{c}},\;N,\;\phi \right).$$

Differentiation with respect to α gives

(19) $$-3\nu \frac{\partial \log\Pi }{\partial \log\left(\lambda /\lambda_{c}\right)}-\frac{\partial \log\Pi }{\partial \log N}+\left(3\nu -1\right)\frac{\partial \log\Pi }{\partial \log\phi }=3\nu .$$

Equation (19) implies that the dimensionless function Π can be represented as

(20) $$\Pi \propto {\left(\frac{\lambda }{\lambda_{c}}\right)}^{\gamma_{1}}N^{\gamma_{2}}\phi^{\gamma_{3}}.$$

Application of Equation (20) to Equation (19) gives

(21) $$-3\nu \gamma_{1}-\gamma_{2}+\left(3\nu -1\right)\gamma_{3}=3\nu .$$

Since we need to determine three unknown exponents, but have a single equation, Equation (21), we need to find additional information. The additional information should be based on correct physics.

#### 2.3.1. Scaling of Modulus

The stress of polymeric fluids is dominantly dependent on chain conformation. In other words, the polymer stress originates from the thermal motion of monomers. If there are $N_{p}$ polymer chains with N monomers, the number of polymer chains per unit volume is given by

(22) $$\frac{N_{p}}{V}=\frac{\phi }{b^{3}N}$$

where V is the volume of the polymer solution. The stress of the polymer solution without entanglement can be assumed as proportional to $k_{B}TN_{p}/V$. We can then choose the scale factor $H_{c}$ by

(23) $$H_{c}^{\left(\mathrm{ue}\right)}=\frac{k_{B}T}{b^{3}}\frac{\phi }{N}.$$

In the case of entangled polymer fluids, the polymer stress should be calculated by the number of the substrands between entanglement. We then obtain

(24) $$H_{c}^{\left(\mathrm{en}\right)}=\frac{k_{B}T}{b^{3}}\frac{\phi }{N_{e}}$$

where $N_{e}$ is the number of monomers in the entangled subchain. In a polymer solution, $N_{e}$ is a function of the polymer volume fraction ϕ.

When self-similarity is applied, we obtain

(25) $$H_{c}^{\left(\mathrm{ue}\right)}\to \alpha^{0}H_{c}^{\left(\mathrm{ue}\right)};\;H_{c}^{\left(\mathrm{en}\right)}\to \alpha^{-1-\chi }H_{c}^{\left(\mathrm{en}\right)}$$

where χ is the exponent, such that

(26) $$N_{e}\to \alpha^{\chi }N_{e}.$$

We will discuss the value of χ later.

#### 2.3.2. Scaling of the Maximum Relaxation Time

By an analogy of chain conformation to diffusion, we can construct the maximum relaxation time $\lambda_{\max}$ as follows:

(27) $$\lambda_{\max}=\frac{R_{\mathrm{diff}}^{2}}{D}$$

where $R_{\mathrm{diff}}$ is the characteristic length scale for diffusion, and D is the diffusion constant. The characteristic length $R_{\mathrm{diff}}$ can be considered as the length scale of relaxation. From the Einstein equation, the diffusion constant D can be represented as

(28) $$D=\frac{k_{B}T}{\zeta }.$$

The friction coefficient ζ is related to the viscosity of the solvent, as follows:

(29) $$\zeta \approx \eta_{s}R_{\zeta }$$

where $R_{\zeta }$ is the characteristic length scale for friction. This is the Stokes equation, where the proportional coefficient 6π has been omitted. Combining Equations (27)–(29), the maximum relaxation time can be defined as

(30) $$\lambda_{\max}=\frac{\eta_{s}}{k_{B}T}R_{\zeta }R_{\mathrm{diff}}^{2}.$$

Since the two length scales $R_{\zeta }$ and $R_{\mathrm{diff}}$ are related to N, b, and ϕ [1], we can find the self-similarity transform of the maximum relaxation time $\lambda_{\max}\to \alpha^{\tau }\lambda_{\max}$. 

By assuming that $H_{c}\to \alpha^{\gamma }H_{c}$ and $\lambda_{\max}\to \alpha^{\tau }\lambda_{\max}$, the self-similarity invariance for Equation (6) can be given by

(31) $$\mu =\frac{\gamma }{\tau }.$$

If γ=0 and τ=0, we need to find additional information to determine μ.

### 2.4. Phenomenology of the Polymer Solution

**Figure 1:**
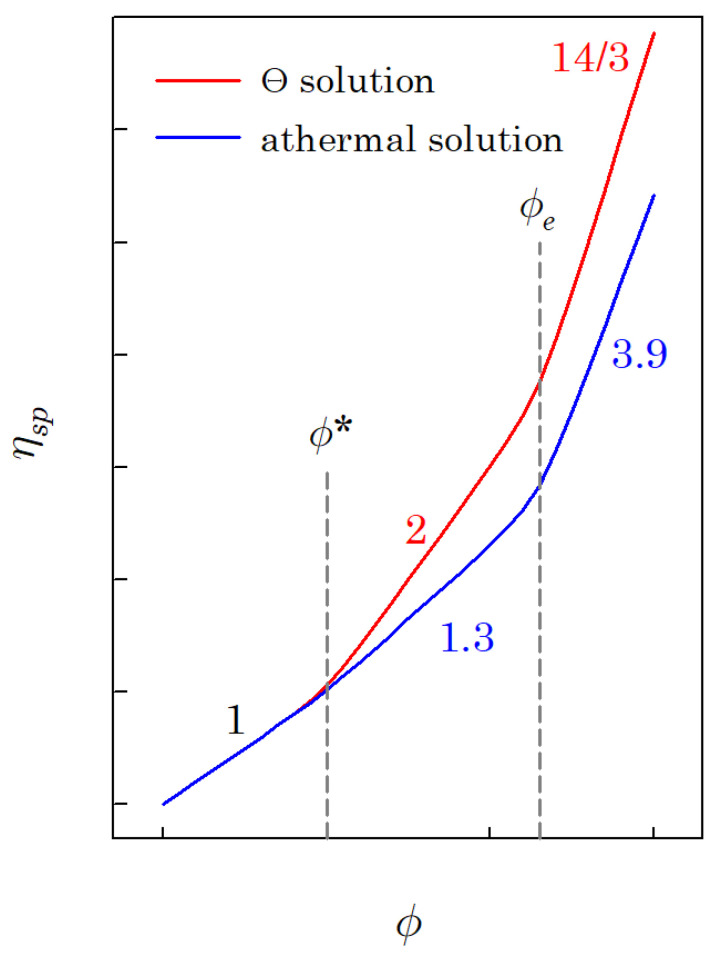
A schematic diagram for specific viscosity of the polymer solution as a function of polymer volume fraction (logarithmic scales).

The specific viscosity of a polymer solution ($\eta_{\mathrm{sp}}$) increases with the polymer volume fraction ϕ, as shown in Figure 1. Figure 1 reveals that there are three concentration regimes. Regime I is the dilute region, where the specific viscosity is linearly proportional to the polymer volume fraction ($\phi <\phi^{*}$; $\phi^{*}$ is the overlap volume fraction). Regime II is the interval of the polymer volume fraction between the overlap and entanglement polymer volume fraction ($\phi^{*}<\phi <\phi_{e}$; $\phi_{e}$ is the entanglement volume fraction). The power-law exponent of regime II is 2 for the Θ solution and about 1.3 for the athermal solution. The last regime is the interval wherein the polymer volume fraction is higher than the entanglement volume fraction ($\phi >\phi_{e}$). The exponent of regime III is 14/3 for the Θ solution and about 3.9 for the athermal solution. If the molecular weight of the polymer is higher than the critical molecular weight ($M>2M_{e}$; $M_{e}$ is the entanglement molecular weight), regime III can be observed, and the entanglement volume fraction can be determined; otherwise, it is hard to observe regime III.

It was reported that, in regime I, the linear viscoelasticity of the polymer solution follows the Zimm model, whereas in regime II, it follows the Rouse model [1]. Additionally, regime III corresponds to the reptational mode. Polymer chains are isolated in regime I, and each polymer chain is indistinguishable in both regimes II and III.

### 2.5. Regime I

Since the polymer chains in regime I are isolated, the radius of gyration of the polymer chain can be considered as the radius of the pervaded volume. The length of relaxational motion can be considered as the radius of the pervaded volume, $R_{\mathrm{diff}}\approx bN^{\nu }$. The length scale of friction $R_{\zeta }$ can also be considered as the radius $R_{\mathrm{diff}}$. Since $R_{\mathrm{diff}}$ is the whole size of the polymer chain, it should represent the maximum length scale of relaxation. Equations (27) and (28) then give

(32) $$\lambda_{\max}^{\left(I\right)}=\lambda_{o}N^{3\nu };\;\lambda_{o}\equiv \frac{\eta_{s}b^{3}}{k_{B}T}.$$

Applying the scaling rule to Equation (32) leads to

(33) $$\lambda_{o}\to \alpha^{3\nu }\lambda_{o};\;\lambda_{\max}^{\left(I\right)}\to \alpha^{0}\lambda_{\max}^{\left(I\right)}.$$

Since no entanglement exists in regime I, we can adopt Equation (23) as the scale factor of modulus. The power-law spectrum of regime I can then be defined as

(34) $$H\left(\lambda \right)=\frac{k_{B}T}{b^{3}}\frac{\phi }{N}{\left(\frac{\lambda }{\lambda_{\max}^{\left(I\right)}}\right)}^{\mu }.$$

However, since both $H_{c}^{\left(\mathrm{ue}\right)}$ and $\lambda_{\max}^{\left(I\right)}$ are the invariants of self-similarity, we cannot determine the exponent μ. We assume that $H\left(\lambda \right)$ is independent of N, corresponding to the molecular weight. We then obtain $\mu =-1/\left(3\nu \right)$.

From Equation (34), we know that the relaxation modulus can be approximated as

(35) $$G\left(t\right)\approx \frac{k_{B}T}{b^{3}}\frac{\phi }{N}{\left[\frac{t}{\lambda_{\max}^{\left(I\right)}}\right]}^{-1/\left(3\nu \right)}\exp\left[-\frac{t}{\lambda_{\max}^{\left(I\right)}}\right].$$

We then obtain

(36) $$G^{\prime }\left(\omega \right)\propto \omega^{1/\left(3\nu \right)};\;G^{\prime \prime }\left(\omega \right)\propto \omega^{1/\left(3\nu \right)}\;\mathrm{for}\;\lambda_{\max}^{\left(I\right)}\omega \gg 1$$

The zero-shear viscosity is given by

(37) $$\eta_{o}=\underset{\omega \to 0}{\lim}\frac{G^{\prime \prime }\left(\omega \right)}{\omega }=\Gamma \left(\frac{1}{3\nu }+1\right)\frac{k_{B}T\phi }{b^{3}N}\lambda_{\max}^{\left(I\right)}\propto \eta_{s}\phi N^{3\nu -1}$$

where $\Gamma \left(x\right)$ is the gamma function. Equation (36) indicates that the zero-shear viscosity is proportional to $N^{3\nu -1}$ and ϕ, which agrees well with the experimental data [1].

**Figure 2:**
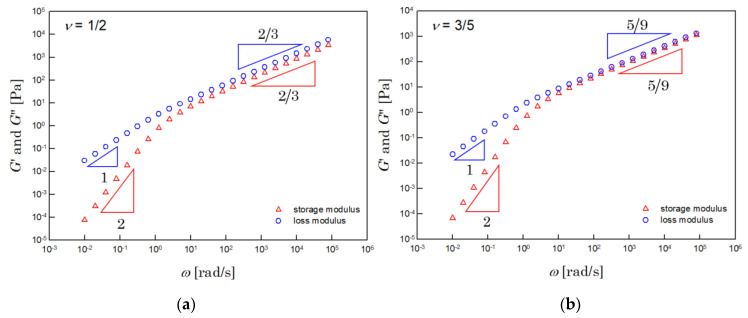
Dynamic moduli of the (**a**) Θ (when υ = 1/2) and (**b**) athermal (when υ = 3/5) solutions. These results were calculated by numerical integration with Equation (7), when $H_{c}=1\mathrm{Pa}$ and $\lambda_{\max}=1s$.

Figure 2 shows the dynamic moduli, numerically integrated by using Equation (7). The left side of Figure 2 shows the results for the Θ solution. The results for the athermal solution are shown on the right. The dynamic moduli obtained were very close to those of the Zimm model.

### 2.6. Regime II

Since the polymer chains cannot be distinguished in regime II, it is difficult to determine any length scale. Thus, we adopted the concept of correlation blob [1]. The correlation blob can be considered as a hypothetical monomer, characterized by the number of real monomers in the blob (g) and size of the blob (ξ). Since the polymer solution was assumed to be filled with the correlation blobs, we have

(38) $$\phi =\frac{gb^{3}}{\xi^{3}}.$$

We made a reasonable assumption that the size of the blob obeys

(39) $$\xi =bg^{\nu }.$$

Note that both ξ and g were determined by the polymer volume fraction ϕ. Combining Equations (38) and (39) gives

(40) $$g=\phi^{-1/\left(3\nu -1\right)};\;\xi =b\phi^{-\nu /\left(3\nu -1\right)}.$$

The relaxation in the blob can be characterized by

(41) $$\zeta_{\mathrm{blob}}\approx \eta_{s}\xi ;\;\lambda_{\mathrm{blob}}\approx \frac{\eta_{s}\xi^{3}}{k_{B}T}$$

where $\zeta_{\mathrm{blob}}$ and $\lambda_{\mathrm{blob}}$ are the friction coefficient and relaxation time of the strand within each correlation blob, respectively.

The friction coefficient of the polymer chain can be considered the sum of the friction coefficient of N/g correlation blobs:

(42) $$\zeta_{\mathrm{chain}}=\zeta_{\mathrm{blob}}\frac{N}{g}.$$

Since the blob is a hypothetical one, it is hard to expect the excluded volume effect for the polymer chains comprising the correlation blobs. The size of the relaxation motion of the chain can be given by

(43) $$R_{\mathrm{diff}}=\xi {\left(\frac{N}{g}\right)}^{1/2}.$$

Equations (42) and (43) give the maximum relaxation time

(44) $$\lambda_{\max}^{\left(\mathrm{II}\right)}=\lambda_{\mathrm{blob}}{\left(\frac{N}{g}\right)}^{2}.$$

With the help of Equations (40) and (41), we obtain

(45) $$\lambda_{\max}^{\left(\mathrm{II}\right)}\to \alpha^{0}\lambda_{\max}^{\left(\mathrm{II}\right)}.$$

Therefore, the maximum relaxation time of regime II is also an invariant of self-similarity. Since entanglement is not involved in regime II, we adopted Equation (23) as the scale factor of modulus. To determine the exponent μ of Equation (6), we assumed that $H\left(\lambda \right)$ is independent of N. We then obtain

(46) $$H\left(\lambda \right)=\frac{k_{B}T}{b^{3}}\frac{\phi }{N}{\left(\frac{\lambda }{\lambda_{\max}^{\left(\mathrm{II}\right)}}\right)}^{-1/2}$$

and it gives

(47) $$G\left(t\right)\approx \frac{k_{B}T}{b^{3}}\frac{\phi }{N}{\left(\frac{t}{\lambda_{\max}^{\left(\mathrm{II}\right)}}\right)}^{-1/2}\exp\left(\frac{t}{\lambda_{\max}^{\left(\mathrm{II}\right)}}\right).$$

This is the relaxation modulus of the Rouse model. 

### 2.7. Entangled Polymeric Fluids

If the molecular weight of the polymer is much higher than the critical molecular weight, regime III can be observed. The solution that belongs to regime III is called an entangled polymer solution. For entangled polymer solutions, Rubinstein and Colby showed that the plateau modulus and reptation relaxation time obey the following scaling rules:

(48) $$G_{e}\left(\phi \right)=\frac{k_{B}T\phi }{b^{3}N_{e}\left(\phi \right)}=\frac{k_{B}T}{b^{3}N_{e}^{\mathrm{melt}}}\left\{\begin{array}{ll} \phi^{3\nu /\left(3\nu -1\right)} & \mathrm{for}\;\mathrm{an}\;\mathrm{athermal}\;\mathrm{solution}\\ \phi^{7/3} & \mathrm{for}\;a\;\Theta \;\mathrm{solution} \end{array}\right.$$

and

(49) $$\lambda_{\mathrm{rep}}\left(\phi \right)=\lambda_{\mathrm{blob}}{\left(\frac{N_{e}}{g}\right)}^{2}{\left(\frac{N}{N_{e}}\right)}^{3}$$

where $N_{e}^{\mathrm{melt}}$ is the number of monomers between entanglement in the molten state [1]. $N_{e}$ is assumed to obey the polymer volume fraction dependence of the correlation number g, as follows:

(50) $$N_{e}\left(\phi \right)\approx N_{e}^{\mathrm{melt}}\left\{\begin{array}{ll} \phi^{-1/\left(3\nu -1\right)} & \mathrm{for}\;\mathrm{an}\;\mathrm{athermal}\;\mathrm{solution}\\ \phi^{-4/3} & \mathrm{for}\;a\;\Theta \;\mathrm{solution} \end{array}\right..$$

It is, thus, obvious that $N_{e}/g=N_{e}^{\mathrm{melt}}$ for any ϕ. The equation of $N_{e}\left(\phi \right)$ for a Θ solution was obtained from the analysis that gives the tube diameter $a\propto \phi^{-2/3}$. With the help of Equation (50), the reptation relaxation time can be rewritten as

(51) $$\lambda_{\mathrm{rep}}\left(\phi \right)=N^{3}\frac{\lambda_{o}}{N_{e}^{\mathrm{melt}}}\left\{\begin{array}{ll} \phi^{3\left(1-\nu \right)/\left(3\nu -1\right)} & \mathrm{for}\;\mathrm{an}\;\mathrm{athermal}\;\mathrm{solution}\\ \phi^{7/3} & \mathrm{for}\;a\;\Theta \;\mathrm{solution} \end{array}\right..$$

Applying the scaling rule to the plateau modulus and reptation relaxation time gives

(52) $$G_{e}\to G_{e}\left\{\begin{array}{ll} \alpha^{1} & \mathrm{for}\;\mathrm{an}\;\mathrm{athermal}\;\mathrm{solution}\\ \alpha^{2/3} & \mathrm{for}\;a\;\Theta \;\mathrm{solution} \end{array}\right.$$

and

(53) $$\lambda_{\mathrm{rep}}\to \lambda_{\mathrm{rep}}\left\{\begin{array}{ll} \alpha^{1} & \mathrm{for}\;\mathrm{an}\;\mathrm{athermal}\;\mathrm{solution}\\ \alpha^{2/3} & \mathrm{for}\;a\;\Theta \;\mathrm{solution} \end{array}\right..$$

Here, we have used $N_{e}^{\mathrm{melt}}\to \alpha^{-1}N_{e}^{\mathrm{melt}}$. Although we expected that $\lambda_{\mathrm{rep}}\to \alpha^{0}\lambda_{\mathrm{rep}}$, Equation (53) does not show invariance. 

If we adopt $H_{c}^{\left(\mathrm{en}\right)}=G_{e}$ and $\lambda_{\max}^{\left(\mathrm{III}\right)}=\lambda_{\mathrm{rep}}$, the exponent μ of Equation (6) can be defined as μ=1 for polymer solutions and melt (ϕ→1). As for the experimental data of monodisperse polymer melts, $G^{\prime \prime }\left(\omega \right)\propto \omega^{-1/4}$ for $\lambda_{\mathrm{rep}}\omega >1$. This means that $H\propto \lambda^{1/4}$. Therefore, the results from self-similarity analysis do not agree with the experimental data. 

We showed that the viscoelasticity of an unentangled fluid (regime I and regime II) agrees with the results from self-similarity analysis. The relaxation of regimes I and II can be represented by the relaxation in a polymer chain, although that of entangled polymeric fluids involve the interaction between the chains (entanglement). We can, thus, conclude that self-similarity analysis holds for polymer systems without entanglement. Note that the power-law spectrum is still effective for polymer melts, but the exponent cannot be determined through self-similarity analysis. 

## 3. Universality of Exponent

### 3.1. Objective

Baumgaertel et al. suggested the power-law spectrum (BSW spectrum) and considered the exponent as a material constant, which can be different for individual polymers. They obtained the exponent from the discrete spectrum by using their numerical algorithm and determined it as 0.22 for polystyrene and 0.42 for polybutadiene [4]. Most molecular theories consider the universality of linear viscoelasticity of monodisperse polymer melts [7,10,11,12]. The material constants of these theories are usually the plateau modulus (scale factor of modulus) and characteristic relaxation time. Cho et al. showed that the experimental data of various monodisperse polymer melts can be superposed on a single curve [5]. Thus, the exponent must be universal.

Although the continuous spectrum is unique, a discrete spectrum is an approximation of the continuous spectrum [13]. Therefore, it is difficult to see that the result extracting the continuous spectrum from a discrete spectrum is perfect, although the IRIS algorithm used by Baumgaertel et al. [4] is one of the most effective algorithms of the discrete spectrum. Due to the ill-posedness of the relaxation spectrum, quite different spectra can show almost the same dynamic modulus.

To check the universality of the exponent, we needed a nonlinear regression that fixes the exponent and determines the scale factors of modulus and relaxation time. According to Baumgaertel et al. [4], we used the following spectrum:

(54) $$H\left(\lambda \right)=\frac{1}{4}G_{e}^{\left(\mathrm{melt}\right)}\left[{\left(\frac{\lambda }{\lambda_{\max}}\right)}^{1/4}+{\left(\frac{\lambda }{\lambda_{E}}\right)}^{-4/5}\right]\;\mathrm{for}\;0<\lambda <\lambda_{\max};$$

(55) $$\lambda_{\max}=\lambda_{E}{\left(\frac{M_{w}}{M_{e}}\right)}^{3.4}$$

where $\lambda_{E}$ and $G_{e}^{\left(\mathrm{melt}\right)}$ are the fitting parameters, $M_{e}$ is the entanglement molecular weight, and $M_{w}$ is the molecular weight. Note that $G_{e}^{\left(\mathrm{melt}\right)}$ is the plateau modulus, which must be a material parameter, and $\lambda_{E}$ is the scale factor of relaxation time. The positive exponent 1/4 is the value from the Doi theory with contour length fluctuation [8]. The negative exponent −4/5 was adopted from the results of Baumgaertel et al. [4].

### 3.2. Regression Method

Since the order of magnitude difference between the three material constants ($\lambda_{E}$, $G_{e}^{\left(\mathrm{melt}\right)}$ and $M_{e}$) was very large, a special nonlinear regression analysis was required. We adopted the Monte Carlo method proposed by Kim et al. [14]. This algorithm is nearly independent of the initial values of parameters, compared to any algorithms based on the gradient. For simplicity purposes, we adopted the values of $M_{e}$ from the literature. Thus, we needed to determine the two material parameters: $\lambda_{E}$ and $G_{e}^{\left(\mathrm{melt}\right)}$.

We generated 10,000 random numbers for $\log\lambda_{E}$ and $\log G_{e}^{\left(\mathrm{melt}\right)}$, in the range $\left(\log\lambda_{E}^{\min},\;\;\;\log\lambda_{E}^{\max}\right)$ and $\left(\log G_{e}^{\min},\;\;\;\log G_{e}^{\max}\right)$. We used $\log\lambda_{E}$ and $\log G_{e}^{\left(\mathrm{melt}\right)}$, instead of $\lambda_{E}$ and $G_{e}^{\left(\mathrm{melt}\right)}$, since the viscoelastic properties vary in logarithmic scale. The plateau modulus estimated with the naked eye can help in determining the boundaries for $\log G_{e}^{\left(\mathrm{melt}\right)}$. Substitution of each random number of Equations (54) and (55) helped in constructing a continuous relaxation time spectrum, thus resulting in dynamic moduli by the numerical integration of Equation (7). Hereon, we will be referring to these as the calculated moduli, $G_{\mathrm{cal}}^{\prime }$ and $G_{\mathrm{cal}}^{\prime \prime }$, compared to the experimental data, $G_{\exp}^{\prime }$ and $G_{\exp}^{\prime \prime }$. We could determine the fitting parameters by comparing the calculated moduli with the experimental data. 

Since $\lambda_{E}$ and $G_{e}^{\left(\mathrm{melt}\right)}$ are the material parameters and fitting parameters, they only depend on the type of polymer. Thus, when we are certain of the dynamic moduli for a few polymers of the same type, but different molecular weights, the coefficient of determination can be given by

(56) $$R^{2}\equiv \frac{1}{2N_{M}}\sum_{m=1}^{N_{M}}\left[\left(1-\frac{{\mathrm{SSE}}_{m}^{\prime }}{{\mathrm{SST}}_{m}^{\prime }}\right)+\left(1-\frac{{\mathrm{SSE}}_{m}^{\prime \prime }}{{\mathrm{SST}}_{m}^{\prime \prime }}\right)\right]$$

where $N_{M}$ is the number of molecular weights. SSE_m_ and SST_m_ represent the sum of square errors and total sum of square for each polymer with a certain molecular weight $M_{m}$, respectively. SSE_m_ and SST_m_, with respect to each modulus, are represented as

(57) $$\begin{array}{l} {\mathrm{SSE}}_{m}^{\prime }=\sum_{\alpha =1}^{N_{\omega }}{\left[\log\frac{G_{\exp}^{\prime }\left(\omega_{\alpha };M_{m}\right)}{G_{\mathrm{cal}}^{\prime }\left(\omega_{\alpha };M_{m}\right)}\right]}^{2};\\ {\mathrm{SSE}}_{m}^{\prime \prime }=\sum_{\alpha =1}^{N_{\omega }}{\left[\log\frac{G_{\exp}^{\prime \prime }\left(\omega_{\alpha };M_{m}\right)}{G_{\mathrm{cal}}^{\prime \prime }\left(\omega_{\alpha };M_{m}\right)}\right]}^{2};\\ {\mathrm{SST}}_{m}^{\prime }=\sum_{\alpha =1}^{N_{\omega }}{\left[\log G_{\exp}^{\prime }\left(\omega_{\alpha };M_{m}\right)-\langle \log G_{\mathrm{cal}}^{\prime }\left(\omega ;M_{m}\right)\rangle \right]}^{2};\\ {\mathrm{SST}}_{m}^{\prime \prime }=\sum_{\alpha =1}^{N_{\omega }}{\left[\log G_{\exp}^{\prime \prime }\left(\omega_{\alpha };M_{m}\right)-\langle \log G_{\mathrm{cal}}^{\prime \prime }\left(\omega ;M_{m}\right)\rangle \right]}^{2} \end{array}$$

where $N_{\omega }$ is the number of frequencies, and $\langle \log G\left(\omega \right)\rangle$ is the mean of $\log G\left(\omega_{\alpha }\right)$. 

Each random number gives a value of $R^{2}$. Moreover, $R^{2}$ will achieve unity when the calculated moduli fit the experimental data perfectly. We provided the reference value for $R^{2}$ and set 0.7 at first. After calculating $R^{2}$ for all the random numbers, we found the minimum and maximum of $\log\lambda_{E}$ and $\log G_{e}^{\left(\mathrm{melt}\right)}$, respectively, among the random numbers corresponding to $R^{2}$ larger than 0.7. They were used as the new ranges for $\log\lambda_{E}$ and $\log G_{e}^{\left(\mathrm{melt}\right)}$. The mean of $R^{2}$ larger than 0.7 worked for the next reference value for $R^{2}$. The more the iterations of this algorithm, the higher was the reference value of $R^{2}$ and narrower was the range of the two fitting parameters. We determined the final values for $\log\lambda_{E}$ and $\log G_{e}^{\left(\mathrm{melt}\right)}$, until the difference between the old and new reference values of $R^{2}$ was under 0.00001.

### 3.3. Results and Discussion

To verify the universality of the exponent in regime III, we used the data of dynamic moduli measured by other researchers (polystyrene (PS) [15], polybutadiene (PBD) [16], polyisoprene (PI) [17], and polymethylmethacrylate (PMMA) [18]). Information regarding these samples is listed in Table 1. All the samples were nearly monodispersed linear polymers. In addition, all of them were fully entangled because $M_{w}/M_{e}>5$.

We analyzed the regression using the Monte Carlo method for the samples in Table 1. Table 2 outlines the values of the determined fitting parameters, as well as the $R^{2}$ values corresponding to them.

**Figure 3:**
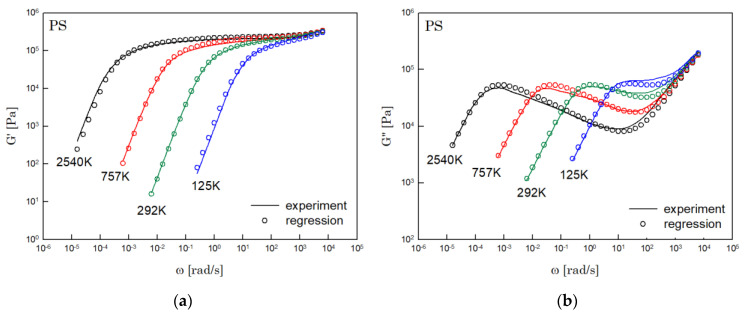
Comparison the regression results (lines) with the experimental data (symbols) of (**a**) storage modulus and (**b**) loss modulus of PS. These experimental data are measured by Schausberger et al. [15].

**Figure 4:**
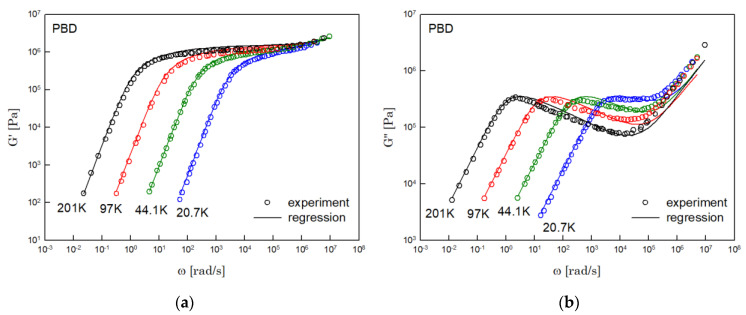
Comparison the regression results (lines) with the experimental data (symbols) of (**a**) storage modulus and (**b**) loss modulus of PBD. These experimental data are measured by Baumgaertel and Winter [16].

**Figure 5:**
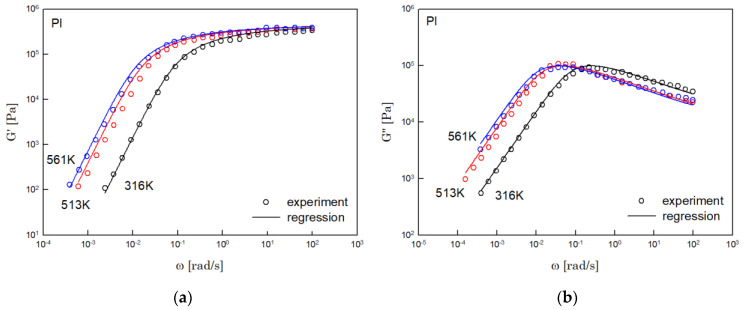
Comparison the regression results (lines) with the experimental data (symbols) of (**a**) storage modulus and (**b**) loss modulus of PI. These experimental data are measured by Person et al. [17].

**Figure 6:**
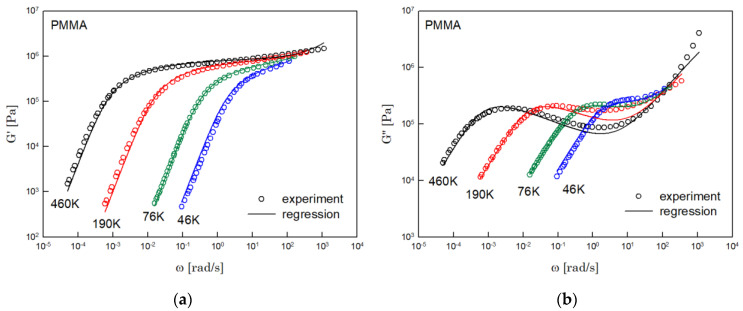
Comparison the regression results (lines) with the experimental data (symbols) of (**a**) storage modulus and (**b**) loss modulus of PMMA. These experimental data are measured by Fuchs et al. [18].

The results of PS, PBD, PI, and PMMA are shown in Figure 3, Figure 4, Figure 5 and Figure 6, in turn. The left side in each figure shows the storage moduli, with the loss moduli shown on the right. Although the exponent of −4/5 was adopted from the material parameter of polystyrene fitting into the BSW spectrum [4], the power-law spectrum of Equation (54) describes the moduli of all the polymers we tested. Regardless of the type of polymer, the qualities of fittings were good, indicating that the universality of exponents holds in regime III and regimes I and II.

## 4. Conclusions

Theoretical models based on molecular theories describe experimental data with only a few adjustable parameters, although they demand complicated calculations. On the other hand, phenomenologically developed models include many fitting parameters, despite simple calculations. The BSW model [4] is a representative phenomenological model for the relaxation time spectrum.

Despite the importance of the concept of self-similarity in polymer physics, there are no studies that have applied the self-similarity to the relaxation time spectrum. Based on the other mathematical results that have dealt with self-similarity, we assumed a relaxation time spectrum as the power-law function represented by two scaling factors: the modulus and maximum relaxation time. The self-similarity transform invariance was used to investigate the power-law spectrum, with respect to the polymer solutions in three regimes: regime I (unentangled, dilute), regime II (unentangled, semidilute), and regime III (entangled, semidilute).

The self-similarity transform invariance of the power-law spectrum holds for regimes I and II. The spectrum showed results consistent with the Zimm and Rouse models. Meanwhile, the power-law spectrum of regime III did not satisfy the invariance, which seems to be due to the interactions caused by entanglement.

However, since the power-law spectrum is still effective for polymer melts, we conducted the further study on various monodisperse polymer melts. It should be noted that the polymer melt could be the extreme case in regime III.

We suggested a power-law formula reflecting the Doi theory [13], with the results of Baumgaertel et al. [4] as the spectrum for the entangled polymer. Since the formula includes two fitting parameters, we adopted the Monte Carlo method [14] to analyze the regression for the experimental data. The good quality of fitting confirmed the validity of the power-law spectrum with the universal exponents in regime III, regardless of the type of polymer, compared to the results of the BSW model [4]. Thus, this model has only two fitting parameters.

## Figures and Tables

**Table 1:** Specifications of samples.

Sample	$M_{w}\;[g/\mathrm{mol}]$	Polydispersity	$M_{e}\;[g/\mathrm{mol}]$
PS	125,000	1.05	15,000
	292,000	1.07	
	757,000	1.07	
	2,540,000	1.05	
PBD	20,700	1.04	1880
	44,100	1.04	
	97,000	1.07	
	201,000	1.27	
PI	316,000	1.04	5200
	513,000	1.06	
	561,000	1.05	
PMMA	46,000	1.03	9200
	76,000	1.06	
	190,000	1.17	
	460,000	1.14	

**Table 2:** Results of the regression analysis for four kinds of polymers.

Sample	$G_{e}^{\left(melt\right)}\;\left[Pa\right]$	$\lambda_{E}\;\left[s\right]$	$R^{2}$
PS	$2.4\times 10^{5}$	$1.6\times 10^{-4}$	0.99
PBD	$1.6\times 10^{6}$	$1.7\times 10^{-7}$	0.98
PI	$4.4\times 10^{5}$	$1.5\times 10^{-5}$	0.98
PMMA	$9.2\times 10^{5}$	$3.4\times 10^{-3}$	0.99

## Data Availability

The data presented in this study are available on request from the corresponding authors.
